# Molecular mechanisms of endothelial dysfunction in Kawasaki-disease-associated vasculitis

**DOI:** 10.3389/fcvm.2022.981010

**Published:** 2022-08-08

**Authors:** Yu Qiu, Yulin Zhang, Yifei Li, Yimin Hua, Yue Zhang

**Affiliations:** Key Laboratory of Birth Defects and Related Diseases of Women and Children of Ministry of Education (MOE), Department of Pediatrics, West China Second University Hospital, Sichuan University, Chengdu, China

**Keywords:** Kawasaki disease, coronary artery disease, inflammation, endothelial cells, molecular mechanisms

## Abstract

Kawasaki disease (KD) is an acute, inflammation mediated vasculitis, mainly affecting in children under five, which is consider as the most common coronary artery disease in children. The injuries of coronary arteries would result in dilation or thrombus formation, bringing great threaten to patients. Endothelium, located in the inner surface of coronary artery, serves as the interface between the circulating inflammatory cells and vascular media or adventitia, which is the first target of inflammatory attacks during early stage of KD. A series of studies have determined vascular endothelial cells damages and dysfunction in KD patients. However, current therapeutic strategy is still challenging. So that it is critical to underline the mechanisms of endothelium injuries. In this review, the role of endothelial cells in the pathogenesis of KD and the therapeutic methods for endothelial cells were systematically described.

## Introduction

Kawasaki disease (KD), also known as mucosa-cutaneous lymph node syndrome, was first reported by Dr. Tomisaku Kawasaki in 1967. Generally, KD mainly attacks children aged under 5 years and demonstrates the highest prevalence among children in Asia. Moreover, the largest patient groups were identified in Japan, South Korea, and China, with a relatively even distribution elsewhere in the world (1). KD has now replaced rheumatic fever as the most common acquired heart disease in pediatric patients worldwide. It is described as an acute febrile extraneous disease that would induce systemic vasculitis mainly targeting small- and medium-sized arteries, which is considered the dominant pathological change accounting for the major adverse effects secondary to KD (2). Acute vasculitis associated with KD may lead to the development of a complex set of coronary artery abnormalities (CAAs). Coronary artery injuries would result in dilation or thrombus formation, both of which endanger patients (3). Dr. Takahashi figured out coronary vasculitis begins 6–8 days after the onset of KD from histopathological examinations, and then the inflammation rapidly invades all layers of the artery, causing significant damage to the structure of the arterial wall, which is the essential cause of CAA development. Monocytes and macrophages have been identified as the major cell types involved in KD-associated arterial inflammatory infiltration. Besides, activated neutrophils were also considered to participate in the initial phase of coronary arteritis. Inflammatory cell infiltration persists for more than 25 days. After that, the accumulation of inflammatory cells gradually reduces, and the pathophysiological process of KD moves to the chronic phase. However, once CAA is initiated in medium- to large-sized arteries, the possibility of recovery diminishes. Arterial dysfunction would persist for a long period in the presence of a large aneurysm or in the event of recanalization after aneurysm embolization (4). As such, it is critical to underline the mechanisms of CAA formation, and several clinical attempts had been made to reduce or avoid the severe situation during the acute phase of KD. Therefore, a series of hypotheses had been raised to explore the association between inflammation and cardiovascular damage.

The endothelium, located in the inner surface of coronary arteries, serves as the interface between the circulation and the vascular media or adventitia, the first target of inflammatory cells during the early stages of inflammatory attacks. Moreover, vascular endothelial cells can secret a variety of pro-inflammatory cytokines, which participate in inducing systemic immune responses and inflammatory activities (5). Several inflammation activation genes, such as NOD1 and NLRP1, contribute to the development of KD and are involved in the regulation of autophagy (6). Endothelial dysfunction due to KD and its association with CAA formation has highlighted the need for further research in recent years (7–9). Endothelial cells also play an important role in the pathogenesis of KD in many aspects as had been pointed out in a consensus of studies. Systemic endothelial dysfunction had been identified in children with a history of KD. Besides, several parameters were identified as independent risk factors for predicting coronary artery lesions, including prolonged febrile periods and increased neutrophil-to-lymphocyte ratios (10). Moreover, some molecular mechanisms were also found to be involved in coronary endothelial dysfunction, such as low-density lipoprotein oxidation and its receptor-mediated signaling activation (11). Qin et al. reported the critical role of autophagy in mediating endothelial dysfunction in KD *via* the co-culture of peripheral blood mononuclear cells (PBMCs) and human coronary artery endothelial cells (HCAECs) (12). Also, non-coding RNAs would always participate in the maintenance of endothelial homeostasis, and miR-197-3p had been detected in KD-associated endothelial damage *via* the regulation of TIMP3 expression (13). In an experimental study, human umbilical cord mesenchymal stem cells could regulate the expression levels of CD54 and CD105 in vascular endothelial cells in KD to suppress the responding inflammation process and reduce endothelial damage, providing a new basis for stem cell therapy for KD (14). Thus, endothelial cells in coronary arteries play an essential role in the outcomes of KD. It is critical to understand the molecular mechanisms of KD-induced endothelial dysfunction and the potential therapeutic strategies that would help maintain endothelial homeostasis. In this review, we focus on the molecular mechanisms of endothelial function in KD and summarize the cutting-edge evidence on endothelial cell therapeutic targets in recent years.

## Endothelial cell dysfunction in Kawasaki disease

The most common but threatening complication associated with KD should be coronary artery lesions (CALs), including coronary artery aneurysm (CAA) or coronary artery dilation (CAD), which would induce coronary artery thrombosis. In severe cases, patients may suffer myocardial infarctions or progress to coronary artery diseases when they get older (15). A study of KD specimens from autopsies (*n* = 32), explanted hearts (*n* = 8), and an incidentally-detected CAA resection by light microscopy (LM) and transmission electron microscopy (TEM) identified three distinct but linked basic vascular pathophysiological processes of KD, including necrotizing arteritis (NA), subacute/chronic (SA/C) vasculitis, and luminal myofibroblast proliferation (16). NA was a synchronous neutrophil process of endothelial cells that occurred within the first 2 weeks of KD. It was considered a self-limiting process that gradually destroyed the homeostasis of the intima, media, and part of the outer membrane of the coronary artery, leading to the development of cystic aneurysms, which would, in turn, lead to aneurysm rupture or thrombosis. It was the dominant cause of KD-induced early death. An increasing number of studies demonstrated that the inflammatory factors induced by KD are accumulated in coronary artery endothelial cells by targeting particular receptors and regulating responding pathways to initiate vasculitis. It was believed that the heterogeneities in the recruitment of chemokines, adhesion molecules, or other molecules on endothelial cells between coronary arteries and other similar-sized arteries should be responsible for the differences in the types of injuries, which decided the specific coronary endothelial dysfunction in KD (17). Li et al. found a significant association between rs2069952, rs9574, and rs1415774 of the endothelial protein C receptor (EPCR) gene and a higher probability of the occurrence of KD in Chinese children (18). The ectodomain of SyndecanSDC-1 could be shed from the cell surface and released into serum, and the shed SDC-1 in serum is regarded as a biomarker for endothelial activation or damage. Serum levels of SDC-1 were significantly higher in patients with KD than in healthy controls and febrile controls (19). As such, the coronary artery endothelial cells serve as the first interface between inflammatory molecules and the vascular wall, and it was important to identify the molecular mechanisms of the role of endothelial cells in KD-associated coronary artery disease.

## Molecular mechanism of vascular endothelial cell injury and dysfunction in KD

### Expression of non-coding RNAs

MicroRNA (miRNA, miR) is a kind of small-molecule RNA with a length of approximately 21 nucleotides, which is involved in the regulation of cell proliferation, apoptosis, inflammation, autoimmunity, and functional maintenance. MiRNAs regulate protein expression *via* the post-transcriptional level by targeting mRNA molecules, mainly affecting the 3′ untranslated regions (3′UTR) (20). In recent years, miRNAs in serum exosomes or coronary artery tissues, including miR-93, miR-186, miR-223, miR-483, and miR-23a have been found to be associated with KD. The downstream signaling of miRNAs provides clues to identifying the molecular mechanisms of KD-induced coronary artery lesions (Table 1).

**Table 1:** Non-coding RNAs regulates vascular endothelial cell injury and maintain in KD.

**miRNA**	**Alteration**	**Target**	**Function**
miR-233	Up	IL6ST	Inhibit STAT3 signal pathway, induce cell injury
miR-233	Up	N/A	Promote cell apoptosis
miR-197-3p	Up	TIMP3	Regulate MMP9, induce cell damage
miR-483	Down	CTGF	Maintain cell homeostasis
miR-27b	Up	SMAD7	Regulate TGF pathway, effect cell migration and proliferation
SOCS2-AS1	Up	MiR-324-5p	Increase cell proliferation and decrease cell apoptosis
miR-324-5p	Down	CUEDC2	Decrease cell proliferation and facilitate cell apoptosis
miR-320a	Up	BMPR1A	Modulate TNF-α production
miR-145-5p	Up	TMEM9B	Regulate the expression of inflammatory cytokines
PINC	Up	N/A	Induce cell apoptosis and inhibit cell proliferation
miR-125a-5p	Up	MKK7	Regulate the Bax/Bcl2 pathway and activates Caspase-3, induced cell apoptosis
miR-186	Up	SMAD6	Induce cell apoptosis
miR-93	Down	VEGF-A	Regulate cell mitogenesis and cell migration

MiRs induce pro-inflammation activities in vascular endothelial cells. Serum miR-223-3p levels were found to be higher in KD patients than in healthy controls. The expression changes in interleukin (IL)-6, intercellular adhesion molecule 1 (ICAM-1), and E-selectin were similar to those of miR-223-3p expression, which increased in the acute stage and reduced in the subacute stage. Also, IL6 was confirmed to be the target gene of miR-223-3p in HCAECs and mouse models, and IL6 would suppress the STAT3 signaling pathway to induce vascular endothelial injury (21). Endothelial microparticles (EMPs) are abundant in circulating blood during inflammation. MiRNAs encapsulated within EMPs had been proven to be involved in the pathogenesis of inflammatory diseases (22). Both miR-320a and miR-145-5p were encapsulated in KD patients with CAL. MiR-320a interacted with *BMPR1A* and correlated with TNF-α expression while miR-145-5p targeted *TMEM9B*, which stimulated IL-6 expression (23). TNF-α was elevated in KD patients, and it induced human umbilical vein endothelial cell (HUVEC) apoptosis. Serum levels of miR-223 were significantly higher in KD patients with CAL than in patients without severe vascular injury, and the serum miR-223 level would decrease after immunoglobulin treatment. However, the miR-233 in serum was mainly secreted by bone marrow-derived blood cells and not transcript in endothelial cells. It was confirmed that miR-233 could be made to enter endothelial cells and promote their apoptosis by co-culturing endothelial cells and macrophages (24).

Besides, miRs had been proven to be involved in several cellular biological processes in endothelial cells subjected to KD. The proliferation of HCAECs could be inhibited by KD serum supplements, and miR-197-3p levels were significantly higher in KD. *TIMP3*, a regulator of matrix metallopeptidase 9 (MMP9), was confirmed as the target of miR-197-3p. Accordingly, the miR-197-3p/TIMP3 axis played a critical role in KD-induced endothelial damage *in vitro* and *in vivo* (13). MMP9 secreted by endothelial cells was thought to take part in CAL formation (25). Endothelial-to-mesenchymal transition (EndoMT) describes the process by which endothelial cells differentiate into mesenchymal cells under various stimulations, and EndoMT was found to be essential for cardiac valve development and involved in a series of cardiovascular diseases such as myocardial infarction, cardiac fibrosis, valve calcification, endocardial elastic fibrosis, atherosclerosis, and pulmonary hypertension (26). A set of spindle-like cells with a high expression of alpha-smooth muscle actin (α-SMA) are thought to be transdifferentiated from endothelial cells to mesenchymal conditions, and the translated cells have great potential in the recruitment of pro-inflammatory cells and induce arterial wall injuries by secreting IL-17, MMPs, and connective tissue growth factor (CTGF). The cells in this category produce disordered collagen, which reduces the structural integrity of the media layer of arteries and contributes to aneurysm formation in KD (27). Also, CTGF was elevated in the coronary arterial wall and serum of KD patients and found to be regulated by miR-483 *via* experiments based on KD serum-treated HUVECs. He et al. revealed that the transcription factor, Kruppel-like factor 4 (KLF4), binds to the promoter region of IGF2-miR-483, up-regulated the expression of miR-483, and maintained coronary artery endothelial cell homeostasis by inhibiting the expression of CTGF (28). While miR-27b was found to be significantly up-regulated in KD serum and HUVECs exposed to KD serum, miR-27b would target *SMAD7* and the Transforming Growth Factor (TGF) pathway, leading to HUVEC migration and proliferation (29). MiR-125a-5p, which is highly expressed in KD, regulates the Bax/Bcl2 pathway and activates Caspase-3 by inhibiting MKK7, resulting in the initiation of HUVEC apoptosis (30). There is a high abundance of miR-186 in serum during KD's acute phase, and the application of KD serum would up-regulate the expression of miR-186 in HUVECs and induce cell apoptosis by targeting *SMAD6* (31). MiR-93 was dysregulated and may be involved in the regulation of vascular endothelial growth factor A (VEGF-A) expression in the pathogenesis of acute KD-induced arteritis (32).

Beyond miRs, long non-coding RNAs (lncRNAs) also contribute to the maintenance of the biological function of coronary artery endothelial cells. Zhao et al. found that lncRNA SOCS2 antisense 1 (SOCS2-AS1) is highly expressed in KD patients' serum and coronary artery tissue, especially in patients with coronary aneurysms. The depletion of SOCS2-AS1 in HUVECs could attenuate cell proliferation and facilitate cell apoptosis. SOCS2-AS1 competitively binds to miR-324-5p to elevate its target gene (*CUEDC2*) expression in the progression of HUVECs in KD (33). lncRNA pregnancy-induced non-coding RNA (PINC) was also involved in the pathogenesis of KD-associated vascular injury (34).

### Inflammatory cell activation

During the acute and subacute phases of KD, significant changes occur among inflammatory cells. A series of studies demonstrated that activated monocytes, neutrophils, and natural killer (NK) cells were significantly associated with coronary artery injuries. So that, the interplay between inflammatory cells and vascular endothelial cells contributes to the formation of CAAs and other related complications. A single-cell RNA-seq study revealed that a subset group of monocytes was involved in KD.

**Figure 1:**
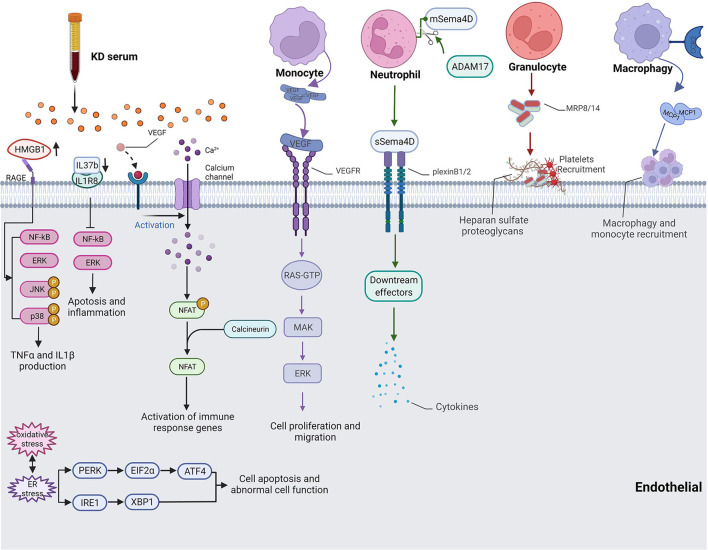
Pathway of cytokine production and inflammatory cell-endothelial cell interaction in KD. HMGB1 up-regulated NF-κB-mediated inflammatory responses and promoted IL1β and TNFα production. IL37 induced apoptosis and inflammation of endothelial cells through the IL-1R8 pathway. VEGF activated Ca2+/NFAT pathway induced inflammation activity by regulating E-selectin, VCAM-1 and MCP-1. Monocyte-produced VEGF also took part in cell migration. Sema4D released by Neutrophil affected endothelial cell cytokine production. Monocytes, macrophage, platelets, and granulocytes recruited to coronary artery and adhesion endothelial cells.

Cell count and neutrophil ratio elevations were major clinical features of KD; thus, the relationship between neutrophils and vascular injuries was a hot topic. Serum Semaphorin 4D (Sema4D) levels were significantly increased in KD patients with CALs. Sema4D had been identified as a pro-inflammatory factor influencing vascular endothelial cell function. Besides, the expression of Sema4D disturbs the cellular function of CD15+ neutrophils and correlates with the shedding proteinase, ADAM17, which interacts with endothelial cells and is associated with cardiovascular disease. Sema4D also promotes the production of cytokines in HCAECs in a dose-dependent manner through the Sema4D-plexin B axis (35) (Figure 1).

Moreover, monocytes had been confirmed to be recruited to the coronary artery and demonstrated more adhesion with endothelial cells. The expression of monocyte chemoattractant protein-1 (MCP1) enhanced the adhesion between monocytes and endothelial cells, which exerts its action by interacting with the C-C chemokine receptor type 2 (CCR2) on the surfaces of monocytes (36, 37) (Figure 1). The expression level of E-selectin was also increased when incubating HCAECs with serum samples of KD with CAL, which led to more endothelium-monocyte interactions. The co-culture of HCAECs with the supernatant of S100A12-activated monocytes induced the expressions of IL6, IL8, ICAM1, and VCAM1 in HCAECs, and this process was mediated by IL1β (38). TNFα or MRP-8/MRP-14, which was secreted by granulocytes, would induce thrombosis and inflammatory responses in patients with acute KD as the consequence of endothelial damage (39) (Figure 1). Neutrophils produced VEGF during the early stages of acute KD while monocytes mainly expressed VEGF from the second week and continued to the fourth week after the onset of KD (Figure 1). The interaction between monocytes and endothelial cells, as well as the secretion of VEGF, influence the proliferation and migration of endothelial cells, resulting in pathological changes in KD-affected coronary artery tissues (40). In a *Lactobacillus casei* cell wall extract (LCWE)-induced macrophages model of KD, the calcium-activated potassium channel, KCa3.1, was highly activated, causing inflammatory reactions in murine coronary artery endothelial cells (MCAECs) (41). DICER1 had been identified to promote the maturation of pre-miRNA in KD-affected platelets after vascular injury, and it contributes to the binding of these platelets to vascular smooth muscle cells (VSMCs), initiating the transport of miRNAs from platelets to VSMCs *via* phagocytosis. Platelet-generated miRNAs inhibited the expression of PDGFRβ in VSMCs after internalization, which prevented vascular smooth muscle cell dedifferentiation and attenuated endothelial repair and damaged tissue healing (42). In another mouse vasculitis model of KD induced by a *Candida albicans* water-soluble extract (CAWS), Miyabe et al. found that Dectin-2 signaling in residual macrophages in the aortic root of the heart induced early CCL2 production and the initial recruitment of CCR2^+^ inflammatory monocytes (iMos) into the aortic root and coronary arteries (43). An elevation in the nuclear factor of activated T cells (NFAT) was recorded in the KD group, and KD serum would activate the signaling of Ca+/NFAT in HCAECs. Then, NFAT induced inflammatory activity by regulating E-selectin, VCAM-1, and MCP-1 (44) (Figure 1).

### Cytokine production

After the activation of inflammatory cells in KD, cytokines would be produced by inflammatory cells and coronary artery endothelial cells that were stimulated by targeting monocytes or neutrophils. Serum-treated HCAECs showed high mitochondrial membrane potentials, increased mitochondrial gene transcription levels, and mitochondrial complex I activity, indicating that oxidative phosphorylation (OXPHOS) had been regulated by cytokines associated with inflammatory cells (45). IL-37 is a member of the IL-1 family and plays an anti-inflammation role in endothelial cells and cardiovascular disease (46). The expression of IL37 decreased in KD serum-treated HUVECs, and exogenous supplementary of IL-37b alleviated KD serum-induced apoptosis and inflammation of endothelial cells through the IL-1R8 pathway. Besides, IL-37b injections remarkably decreased VCAM-1 expression and the infiltration of macrophages and neutrophils in a CAWS-induced KD mouse model (47) (Figure 1). Anzai et al. found that CAWS mediates IL-1β and NLRP3 inflammasome activation through the Dectin-2/Syk/JNK/NF-κB pathway and the Dectin-2/Syk/JNK/mitochondrial Reactive oxygen species (mtROS) pathway, which both participate in KD vasculitis (48). High mobility group box B1 (HMGB1) is a molecular pattern molecule associated with extracellular damage and a key regulator of autophagy (49). HMGB1 is released by endothelial cells when exposed to microorganisms, pathogens, and endogenous inflammatory factors (50). HMGB1 and its receptors, RAGE, TLR2, and TLR4, were up-regulated in KD serum-treated HCAECs; however, only RAGE could decrease after prednisolone administration. They also up-regulated NF-κB-mediated inflammatory responses in KD and promoted IL1β and TNFα production to cause endothelial cell injury (51) (Figure 1).

### Reactive oxygen species accumulation

Excessive reactive oxygen species (ROS) represent endothelial dysfunction, which leads to the progression of coronary artery inflammation or dysfunction. Once prolonged or excessive perturbations exist, the unfolded protein response might trigger intracellular signal cascades and induce oxidative stress, inflammation, and apoptotic responses (52). Bollmann et al. found that systemic inflammation caused by tristetraprolin deficiency leads to endothelial dysfunction. Oxidative stress, especially increased Nox2 activity, promoted the development of atherosclerosis in models of systemic inflammatory diseases (53). Oxidative and ER stress was identified to be involved in KD-induced damages in HCAECs via KD serum stimulation. ROS production and its related molecules, including ATF4, p-EIF2α, p-PERK, XBP1, p-IRE1, HSP90B1, HSPG2, DNAJC3, P4HB, and VCP, were increased in KD patients and decreased by berberine treatment (52) (Figure 1). In giant cell arteritis, immature neutrophils migrate from both lumen and capillaries to adhere to the elastic lamina and release ROS in an inflammatory microenvironment for a prolonged duration, leading to aggressive protein oxidation and the disruption of the permeability of the endothelial barrier in an *in vitro* experimental system (54). Martin et al. found that endothelial cell-derived microparticles expressing VCAM-1 or C4d increase in KD patients and cause endothelial dysfunction by releasing substances such as ROS and cytokines (55). Hypoxia-inducible factor (HIF) signaling had been considered a protective mechanism against ROS in endothelial cells. Ehling et al. found that the B55α/PP2A complex restrained PHD-2's activity to promote endothelial cell survival in a HIF-dependent manner and dephosphorylated p38 (56). During angiogenesis, endothelial progenitor cells are recruited from the bone marrow and differentiate *in situ* into mature endothelial cells under the influence of NO produced by eNOS activation (57). NADPH oxidase (NOX), a primary cause of oxidative stress in the vasculature, contributed significantly to endothelial dysfunction in the microcirculation under excessive ROS exposure, subsequently disrupting nitric oxide (NO) signaling in KD. The product of this reaction, peroxynitrite, was a kind of powerful oxidant and could exacerbate vascular dysfunction by causing further damage to lipids, proteins, and DNA, uncoupling endothelial nitric oxide synthase (eNOS), and diminishing smooth muscle responses to NO (58).

### Lipid oxidation

Oxidized low-density lipoprotein (oxLDL), which is derived from low-density lipoprotein (LDL), is a specific lipid metabolite produced under oxidative stress and the most active component of lipoproteins that promotes the development of atherosclerosis. As a specific receptor of oxLDL, lectin-like-oxLDL receptor-1 (LOX-1) was the only receptor that could be released from the cell surface to form a soluble scavenger molecule. LOX-1 is mainly expressed in vascular endothelial cells of coronary arteries, macrophages, lymphocytes, and dendritic cells. Previous studies showed that the interaction between oxLDL and LOX-1 was a key mechanism for endothelial cell injury (59). A study conducted on 80 children with KD, 20 febrile children, and 20 healthy children revealed that the plasma oxLDL concentration and LOX-1 mRNA expression in PBMCs were significantly higher in children with KD in the acute phase, especially when associated with CAL formation (11).

### Targeting endothelial cell treatment in KD

As endothelial cells serve as a major target during KD, endothelial cell injuries play a critical role in the development of adverse complications, especially for KD-associated coronary artery disease. Thus, attempts have been made to attenuate or reverse endothelial cell damage in response to excessive inflammatory activity. Atorvastatin has been shown to help maintain endothelial cell homeostasis and suppress vascular inflammation and has been identified as a potential new candidate treatment for KD. Atorvastatin could activate KLF4, reducing CTGF production of KD-injured endothelial cells. Besides, atorvastatin showed great potential to be an additional alternative to the standard treatment by significantly reducing CTGF levels in patients (28). A phase I/IIa dose-escalation study of atorvastatin in KD patients with CAA validated the safety and pharmacokinetic data of atorvastatin (60). Intravenous immunoglobulin (IVIG), which is somehow efficient in most patients, has been used as the first-line standard treatment of KD. The addition of prednisolone to intravenous immunoglobulin for acute KD may ameliorate HMGB-1-mediated inflammation in KD-induced vasculitis (51). In a HCAEC cell model of KD, the addition of corticosteroids to standard IVIG therapy suppressed cellular Caspase3/7 activity and inhibited cell apoptosis. It can also inhibit the release of HMGB1 and reduce the expression of three HMGB1-mediated inflammatory cytokines, TNF-α, IL-1α, and IL-1β, which is of great benefit to the clinical treatment of patients with severe KD (61). Resveratrol inhibits TNF-α-induced ICAM-1 expression via the activation of autophagy (62). 1α,25-dihydroxy vitamin D3 (1-25(OH)_2_-VitD3) has an inhibitory effect on TNF-α-induced E-selectin expression, inhibits TNF-α-induced NF-κB activation in HUVECs, and modulates the inflammatory response in KD vasculitis (63). 1-25(OH)_2_-VitD3 also inhibits TNF-induced ICAM-1, VCAM-1, IL-6, and IL-8 expression in HCAECs (64). Cyclosporine is an immunosuppressant that blocks calcineurin, a downstream molecule of the Ca^2+^/NFAT pathway that inhibits nuclear translocation of NFAT-regulating genes, thereby mediating immunosuppression. The researchers studied the efficacy of cyclosporine A in the treatment of IVIG resistant and refractory Kawasaki disease. It is speculated that from a pharmacogenomics perspective, the study of new Ca^2+^/NFAT pathway inhibitors may be promising in treatment (44).

Moreover, with the rapid development of gene therapy, it allows to generate specific RNAi, gene overexpression, knock-down strategies for endothelial cells with adeno-associated virus (AAV) or particular nanoparticles. The current gene therapy process much shorten the develop duration for a newly invented medication, and expanded the capability to handle specific genes expression. As the above content mentioned, several miRs have involved in KD related coronary artery disease, the AAV vector presented great efficient to target abnormal miRs and provide protective role in reducing endothelial injuries (65).

## Conclusion

It has been 50 years since KD proposed for the first time. Although the etiology and the initiating factor is always not clear, but for KD pathology and coronary arterial vasculitis of research to improve our essential understanding of KD. At present, it is generally accepted that KD may be one or more uncertain infection factor in genetically susceptible individuals induced intense inflammation host response. Vascular endothelial cell inflammation caused by KD has been persistent since the beginning of the disease. Therefore, it is of great significance to discover serum biomarkers with diagnostic significance for endothelial cell injury in the early stage of the disease and to carry out treatment for vascular endothelial cell injury in the near future. Further research should focus on cytogenetics and molecular biology, so that more effective targeted drugs can be used in clinic to improve the prognosis of children with KD.

## Author contributions

YueZ and YL conceived of the presented idea. YQ, YulZ, YL, and YueZ summarized the reference and draft the manuscript. YueZ organized the figure with online free material. YueZ and YH supervised the project and contributed equally to the final version of the manuscript. All authors contributed to the article and approved the submitted version.

## Funding

All phase of this study was supported by Central Government Funds of Guiding Local Scientific and Technological Development for Sichuan Province (2021ZYD0105) and Natural Science Foundation of China (81700360). The funders had no role in study design, data collection and analysis, decision to publish, or preparation of the manuscript.

## Conflict of interest

The authors declare that the research was conducted in the absence of any commercial or financial relationships that could be construed as a potential conflict of interest.

## Publisher's note

All claims expressed in this article are solely those of the authors and do not necessarily represent those of their affiliated organizations, or those of the publisher, the editors and the reviewers. Any product that may be evaluated in this article, or claim that may be made by its manufacturer, is not guaranteed or endorsed by the publisher.
